# Incidental detection of primary hepatocellular carcinoma on 18F-prostate-specific membrane antigen-1007 positron emission tomography/computed tomography imaging in a patient with prostate cancer: A case report

**DOI:** 10.1097/MD.0000000000022486

**Published:** 2020-10-09

**Authors:** Hongguang Zhao, Yinghua Li, Sen Hou, Yuyin Dai, Chenghe Lin, Songbai Xu

**Affiliations:** a Department of Nuclear Medicine; b Department of Neurosurgery, The First Hospital of Jilin University, Changchun, China.

**Keywords:** hepatocellular carcinoma, positron emission tomography-computed tomography, prostate cancer, prostate-specific membrane antigen

## Abstract

**Rationale:**

Prostate-specific membrane antigen positron emission tomography-computed tomography (^18^F-PSMA-1007 PET/CT) imaging is an emerging method for the diagnosis of prostate cancer (PC), but its efficiency in detecting other accompanying diseases has rarely been investigated.

**Patient concerns:**

A 77-year-old man presented with a complaint of bone pain throughout his entire body lasting for 2 weeks. Routine preoperative whole-body bone scanning revealed multiple osteogenic metastases. His alpha-fetoprotein and prostate-specific antigen levels were 108.2 ng/mL and 53.32 ng/mL, respectively. ^18^F-PSMA-1007 PET/CT imaging revealed high tracer uptake in the primary lesion in the liver and the peripheral zone of the prostate.

**Diagnoses:**

Due to the results from imaging and pathological examinations, a diagnosis of PC with multiple bone metastases accompanied by primary hepatocellular carcinoma was made.

**Interventions:**

Taking into consideration the patient's age, interventional therapy was performed for the liver lesion, whereas the prostate and bone lesions were treated with endocrine therapy.

**Outcomes:**

The patient recovered well and was discharged uneventfully postoperatively. The patient was also doing well at the 6-month follow-up.

**Lessons:**

PSMA-PET/CT imaging results must be interpreted cautiously when the uptake of PSMA increases in a single lesion instead of the most common sites of PC metastasis. Pathological examination of the suspected lesions is also recommended.

## 1 Introduction

Due to advancements in technology, several of the radioactive tracers widely used in recent years including Fluorine and Gallium (^18^F and ^68^Ga) can help in the detection of prostate cancer (PC). Prostate-specific membrane antigen positron emission tomography-computed tomography (^68^Ga-PSMA-11 PET/CT) imaging has emerged as the latest technique offering high precision for identifying recurrent PC even with a low prostate-specific antigen (PSA) level.^[1]^^18^F has a longer half-life than ^68^Ga (110 minutes vs 68 minutes) with only a few limitations related to the generator capacity. The effects of several ^18^F-labeled ligands have been evaluated in preliminary clinical investigations.^[2,3]^ Based on the low background activity in the urinary tract, ^18^F-PSMA-1007 has the potential to be the next clinical routine utility.^[3]^

In the present study, we report a rarely seen case of ^18^F-PSMA-1007 PET/CT imaging of PC accompanied by primary hepatocellular carcinoma (HCC).

## 2 Case

**Figure 1:**
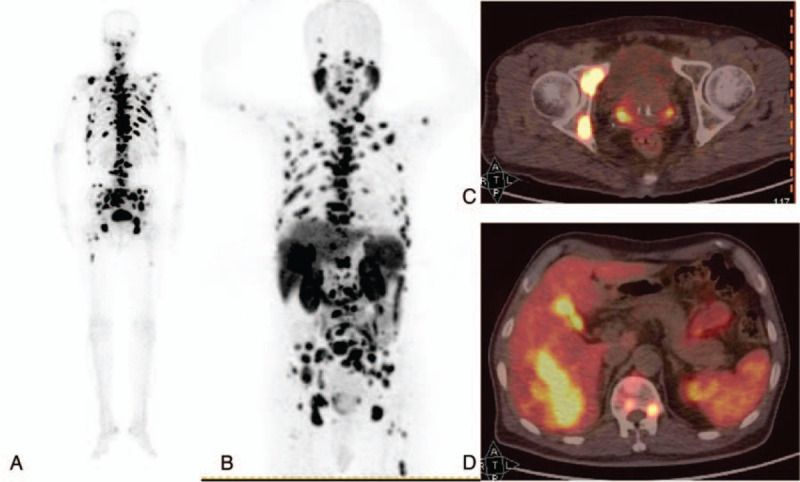
^99m^Tc-MDP whole-body bone scanning and ^18^F-PSMA-1007 PET/CT imaging of the patient (gender: male, age: 77 years) with PC accompanied by primary HCC. (A) ^99m^Tc-MDP imaging suggests multiple bone metastases in the whole body; (B) ^18^F-PSMA-1007 PET MIP imaging suggesting multiple hypermetabolic lesions in the liver, bone, prostate, and lymph nodes; (C) ^18^F-PSMA-1007 PET/CT fusion imaging suggesting localized high metabolite levels within the bilateral peripheral lesions of the prostate with an SUVmax of 11.7; D,^18^F-PSMA-1007 PET/CT fusion imaging suggesting a high metabolite lesion in the right lobe of the liver with an SUVmax of 27.5. ^18^F = Fluorine, HCC: hepatocellular carcinoma, PC: prostate cancer, PET/CT: positron emission tomography/computed tomography, PSMA: prostate-specific membrane antigens, SUVmax: maximum standardized uptake value.

**Figure 2:**
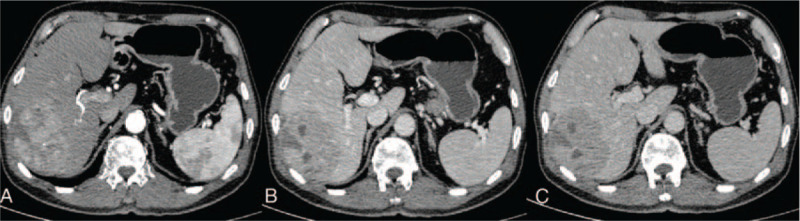
Total abdominal 3-phase enhanced CT imaging of the patient (gender: male, age: 77 yr) suffering from PC accompanied by primary HCC. (A) In the arterial phase, the lesions look unevenly enhanced and the blood supply artery is visible. (B and C) The venous phase and the equilibrium phase, the degree of lesion enhancement is reduced with some lipid components, suggesting that the lesion was derived from liver cells. CT = computed tomography, HCC: hepatocellular carcinoma, PC: prostate cancer.

We report the case of a 77-year-old male patient who was suspected of having multiple bone osteogenic metastases potentially derived from PC in January 2019. The patient reported no history of hepatitis and complained of bone pain throughout his entire body lasting for 2 weeks. Technetium 99m-methyl diphosphonate (^99m^Tc-MDP) single-photon emission-CT revealed multiple bone osteogenic metastases throughout the body (Fig. 1). This presentation was strongly suspected to indicate the presence of a prostate malignancy. For confirmation, ^18^F-PSMA-1007 PET/CT imaging was performed (Fig. 1). The results showed high metabolite levels within the range of the bilateral peripheral lesions of the prostate. The prostate lesions showed a maximum standardized uptake value (SUVmax) of 11.7 and a bone SUVmax of 28.4, with a striking increase in metabolism in the right lobe of the liver (a mass of about 7.8 cm), which exhibited an SUVmax of 27.5. These results further confirmed that the patient suffered from PC with multiple bone metastases. Tumor marker analysis revealed that the level of alpha-fetoprotein was 108.2 ng/mL and the level of PSA was 53.32 ng/mL. No abnormality in any other marker was detected. We sought to determine whether the patient suffered from liver metastasis that originated from PC or PC combined with primary liver cancer. Abdominal 3-phase enhanced CT scans showed uneven enhancement in the arterial phase as well as reduced enhancement in the venous and equilibrium phases, indicating the possibility of HCC (Fig. 2). The patient underwent a puncture biopsy for the liver and prostate lesions. The pathology report suggested that the liver and prostate lesions were HCC and PC, respectively. Thus, the patient was diagnosed with PC with multiple bone metastases accompanied by primary HCC. The liver lesion was treated with interventional therapy while the prostate and bone lesions were treated with endocrine therapy as surgery was ruled out considering the age of the patient. The patient was found to be doing well at the 6-month follow-up. The patient provided written informed consent with his signature. We carried out the study after obtaining approval from the Ethics Committee at the First Hospital of Jilin University.

## 3 Discussion

Several studies have been conducted to confirm the diagnostic accuracy of ^18^F-PSMA-1007 PET/CT imaging for PC compared with traditional PET/CT imaging. Afshar-Oromieh et al recently reported a cohort study with 319 patients involving ^68^Ga-PSMA PET/CT imaging.^[1]^ The results showed that the sensitivity was 76.6%, the specificity was 100%, the negative predictive value was 91.4%, and the positive predictive value was 100%. A meta-analysis conducted on 1309 patients indicated that the sensitivity and specificity of ^68^Ga-PSMA-PET/CT imaging were both 86%.^[4]^ Several studies have been conducted with a focus on evaluating the effect of ^68^Ga-PSMA imaging accuracy for the diagnosis of PC. However, ^18^F produced by a circular accelerator has higher activity, a longer half-life, and higher physical spatial resolution compared with the low-activity ^68^Ga extracted from a ^68^Ge/^68^Ga generator. ^18^F also has advantages over ^68^Ga when the lower excretion of PSMA-1007 in the urinary system reduces interference from the bladder in the detection of pelvic lesions. Rahbar K performed ^18^F-PSMA imaging on 100 PC patients who were suspected of biochemical recurrence and found that the cumulative detection rates for PSA levels ≤0.5, 0.51 to 1.0, 1.1 to 2.0, and >2.0 ng/mL were 86%, 89%, 100%, and 100%, respectively.^[5]^ Another meta-analysis performed on different studies involving 645 patients showed the cumulative detection rate of ^18^F-PSMA PET/CT in biochemical recurrent PC was 81% with a 95% confidence interval (CI) of 71% to 88%. The cumulative detection rates for PSA <0.5 ng/mL and PSA >0.5 ng/mL were 49% (95% CI: 23%–74%) and 86% (95% CI: 78%–93%), respectively.^[6]^ Therefore, PSMA-PET/CT has already gained recognition in the technology domain for the diagnosis of noninvasive PC.

There are many scholarly articles in recent years confirming that ^68^Ga-PSMA detection not only occurs in PC but also in nonprostatic neovascularization or benign tissues.^[7]^ PSMA is a type II transmembrane glycoprotein first discovered in the human PC cell line LNCaP and is widely present in prostate tissue. However, the expression of PSMA is more than 100-fold higher in PC than in normal tissues.^[8]^ The term PSMA is used to indicate the expression of the protein with high specificity in prostate tissue, but this membrane-bound dinuclear zinc-metallopeptidase is also called glutamate carboxypeptidase II, N-acetylated-alpha-linked acidic dipeptidase, and folate hydrolase. The protein is widespread across different tissues in the human body including prostatic acinar epithelium, renal tubules, small intestine, astrocytes, and Schwann cells of the nervous system. However, the expression of PSMA is lower in the heart, pancreas, bladder, skin, breast, liver, lung, colon, and testicular tissues. The apical and luminal surfaces of neovascular endothelial cells in the tumor neovascular system, as well as in some normal hyperplasia tissues like the endometrium, heart valve injury, pleural lesions, and keloids have high PSMA expression.^[9]^ PSMA uptake has been randomly reported in cases of colon, esophageal, thyroid, lung, and liver cancers, intrahepatic cholangiocarcinoma, renal cell carcinoma, brain tumors, sarcoidosis, Paget bone disease, rib fractures, and so on.^[10–12]^ Kesler M et al performed ^68^Ga-PSMA PET/CT imaging on 41 lesions in 7 patients suspected of having HCC^[13]^ and reported that ^68^Ga-PSMA positivity was detected in 36/37 tumor lesions. They also found that none of the 4 regenerative nodules examined showed an increase in ^68^Ga-PSMA uptake. The detection rate of lesions with ^68^Ga-PSMA was higher when compared to that achieved with ^18^F-fluorodeoxyglucose (^18^F-FDG; 10 lesions were detected). This evidence indicates that HCC could be present in regions with ^68^Ga-PSMA uptake as well.

There are limited reports discussing the high PSMA uptake in patients with PC accompanied by any other primary malignancy. Osman MM conducted a study on the efficacy of ^68^Ga-PSMA PET/CT imaging for 764 patients between 2013 and 2016.^[14]^ Only 0.7% (5/764) of the patients with high PSMA uptake lesions had a second primary tumor and these included lung adenocarcinoma, squamous cell carcinoma, diffuse large B lymphoma, papillary thyroid carcinoma, and oral squamous cell carcinoma. It was also found that 24 patients with benign lesions showed high PSMA uptake including laryngological physiologic uptake, thyroid Hurthle cell adenoma, hepatic hemangioma, and atypical meningioma.

^18^F-PSMA PET/CT imaging for PC and primary HCC in a single patient is rarely reported. In fact, our study represents the first case in which the patient had a single lesion in the liver with high PSMA uptake, which was suspected to be metastasis from PC but ultimately found to be HCC. Several studies in addition to ours are in agreement about the high specificity of PSMA-PET/CT imaging for PC patients. However, the results of PSMA-PET/CT imaging must be interpreted cautiously when the uptake of PSMA increases in a single lesion instead of the most common sites of PC metastasis. We recommend examining the suspected lesions pathologically as needed.

## Author contributions

Hongguang Zhao made substantial contributions to the conception and design of the research, Yinghua Li and Sen Hou carried out data collection and analysis, Hongguang Zhao, Yuyin Dai, Chenghe Lin, and Songbai Xu wrote the paper, Hongguang Zhao, Yinghua Li, Sen Hou, Yuyin Dai, Songbai Xu, and Chenghe Lin edited the manuscript and provided critical comment. All authors read and approved the final manuscript.

## Abbreviations

Abbreviations: ^18^F = fluorine, ^18^F-FDG = ^18^F-fluorodeoxyglucose, ^68^Ga = gallium 68, CI = confidence interval, HCC = hepatocellular carcinoma, PC = prostate cancer, PET/CT = positron emission tomography/computed tomography, PSMA = prostate-specific membrane antigen, PSA = prostate-specific antigen, SUVmax = maximum standardized uptake value.
